# Isoflurane depresses hippocampal CA1 glutamate nerve terminals without inhibiting fiber volleys

**DOI:** 10.1186/1471-2202-7-5

**Published:** 2006-01-12

**Authors:** Bruce D Winegar, M Bruce MacIver

**Affiliations:** 1 Stanford Neuroscience Program and Neuropharmacology Laboratory, Department of Anesthesia, Stanford University School of Medicine, Stanford, CA 94305-5117, USA

## Abstract

**Background:**

Anesthetic-induced CNS depression is thought to involve reduction of glutamate release from nerve terminals. Recent studies suggest that isoflurane reduces glutamate release by block of Na channels. To further investigate this question we examined the actions of isoflurane, TTX, extracellular Ca^2+^, CNQX and stimulus voltage (stim) on glutamate-mediated transmission at hippocampal excitatory synapses. EPSPs were recorded from CA1 neurons in rat hippocampal brain slices in response to Schaffer-collateral fiber stimulation.

**Results:**

Isoflurane (350 μM; 1 MAC) reversibly depressed EPSP amplitudes by ~60% while facilitation increased ~20%. Consistent with previous studies, these results indicate a presynaptic site of action that involves reduced excitation-release coupling. EPSPs were depressed to comparable levels by TTX (60 nM) or lowered stim, but facilitation was not changed, indicating a simple failure of axonal conduction. Similarly, partial antagonism of postsynaptic glutamate receptors with CNQX (10 μM) depressed EPSP amplitudes with no change in facilitation. However, EPSP depression by low external Ca^2+ ^(0.8 mM) was accompanied by an increase in facilitation comparable to isoflurane. Isoflurane depression of EPSP amplitudes could also be partly reversed by high external Ca^2+ ^(4 mM) that also decreased facilitation. Isoflurane or low Ca^2+ ^markedly reduced the slopes of fiber volley (FV)-EPSP input-output curves, consistent with little or no effect on FVs. By contrast, TTX didn't alter the FV-EPSP curve slope, indicating that EPSP depression resulted from FV depression. FVs were remarkably resistant to isoflurane. Somatic spike currents were unaffected by 350 μM (1 MAC) isoflurane as well. The EC_50 _for isoflurane depression of FVs was ~2.8 mM (12 vol. %; 8 MAC).

**Conclusion:**

Isoflurane appears to depress CA1 synapses at presynaptic sites downstream from Na channels, as evident by the increased facilitation that accompanies EPSP depression. Fiber volleys did not exhibit depression by isoflurane, as has been reported for other brain regions.

## Background

A longstanding controversy in the anesthesia literature concerns whether general anesthetics exert their actions primarily by depression of synaptic transmission or by blocking nerve conduction like local anesthetics. Thesleff [1] first proposed that general and local anesthetics share a common mechanism to block Na conductance [cf. [2-4]]. Alternatively, other early studies yielded evidence that volatile anesthetics preferentially depress synaptic potentials rather than axonal conduction [5,6]. At present, most studies of volatile anesthetic molecular targets are directed toward synaptic ligand-gated ion channels, as these sites appear more sensitive to volatile anesthetics than voltage-gated channels [7]. However, other studies have shown that volatile anesthetics depress axonal conduction or block voltage-gated Na channels. Berg-Johnsen and Langmoen [8] reported that 1 MAC isoflurane (1.5 vol. %) produced an 18% depression of fiber volley amplitudes recorded from small unmyelinated fibers in the hippocampal CA1 region. More recently, Mikulec et al. [9] reported that 1 MAC halothane depressed Schaffer-collateral axon fiber volleys by 18%. These findings and others support a presynaptic action of volatile anesthetics to depress axonal excitability.

Glutamatergic synapses are of particular interest with regard to anesthetic mechanisms. These excitatory synapses are distributed across the neural axis and are especially abundant in telencephalic structures such as hippocampus and cortex [10,11]. Volatile anesthetics have long been recognized to depress glutamatergic neurotransmission, with evidence for both pre- and postsynaptic sites of action, including actions to depress excitability [12-14]. Some specialized glutamatergic synapses have enabled investigators to specifically look at anesthetic actions in electrically isolated presynaptic structures. Wu et al. [15] reported that isoflurane at clinical concentrations produced small reductions in spike amplitudes in the calyx of Held. The nonlinearities in coupling of excitability to Ca^2+ ^influx and vesicle fusion produced a proportionally larger action of isoflurane to reduce transmitter release. Ouyang et al. [16] found that isoflurane inhibited Na channels in isolated neurohypophyseal nerve terminals. In addition, Sandstrom [17] reported that isoflurane reduced presynaptic excitability in the phylogenetically distant larval *Drosophila *glutamatergic neuromuscular junction – an effect that is also consistent with Na channel inhibition.

We sought to address the question of whether isoflurane inhibits voltage-gated Na channels to depress glutamatergic neurotransmission in the hippocampus. Earlier investigations found that isoflurane depressed glutamate release from synaptosomes by inhibiting voltage-gated Ca channels [18], although P/Q and N-type Ca channels don't appear to be directly targeted [19,20]. Volatile anesthetics may directly act on presynaptic Na channels to depress neurotransmission. Studies in the hippocampus will help to ascertain whether volatile anesthetics generally inhibit Na^+ ^conductance in the CNS or if this action is more specifically associated with the specialized synapses where it has been best documented [15,16].

## Results

### Tetrodotoxin depresses neurotransmission without effect on facilitation

**Figure 1:**
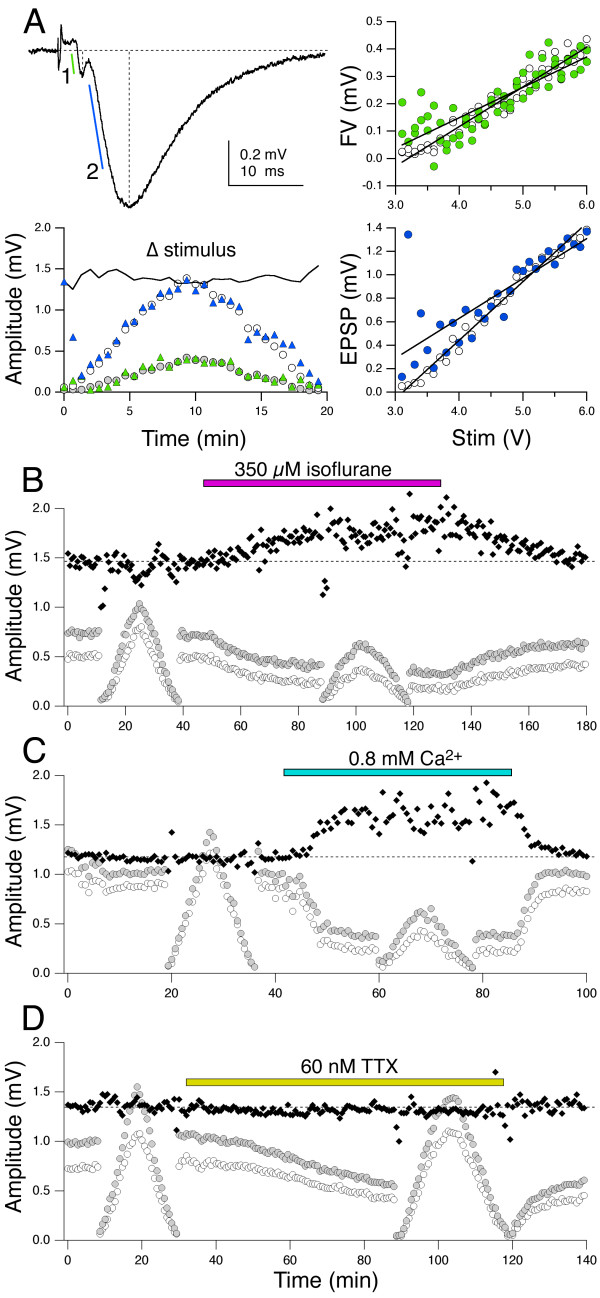
**Enhancement of facilitation by isoflurane and low external Ca^2+ ^but not by TTX**. *A*, (*top left*) an averaged extracellular recording showing two methods that were used for signal parameter measurement: a minimum-to-baseline (MTB) algorithm was used to measure FV and EPSP amplitudes (dashed lines); a *dV/dt *(slope) algorithm was used to measure the initial slope of FVs (1) and EPSPs (2). *A*, (*top right*) comparison of slope and amplitude measures of FVs as a function of stimulus voltage. Open circles are FV amplitudes measured by MTB (r^2 ^= 0.99; n = 60). Green circles are FV slope measurements that were scaled to match the amplitude data (r^2 ^= 0.85; n = 60). Each data set was fit by linear regression (solid lines). *A*, (*lower right*) comparison of slope and amplitude measures of EPSPs as a function of stimulus voltage. Open circles are EPSP amplitudes (r^2 ^= 0.97; n = 30). Blue circles are EPSP slope measurements that were scaled to match the amplitude data (r^2 ^= 0.83; n = 30). Solid lines represent fits by linear regression. *A*, (*lower left*) an experiment showing changes in FV amplitudes (gray circles) and scaled slopes (green triangles) as stimulus voltage was increased and decreased. EPSP amplitudes (open circles) are plotted together with scaled EPSP slopes (blue triangles). Although the stimulus voltage was changed over a 3–6 V range there was no change in facilitation (solid black line). *B*, Isoflurane depressed EPSPs (white and gray circles) and increased facilitation (black diamonds). *C*, Low external Ca^2+ ^(0.8 mM) depressed EPSPs and increased facilitation. *D*, 60 nM TTX depressed EPSPs while facilitation remained unchanged. Note that changes in the stimulus voltage in all three experiments (appearing as ramps in EPSP amplitudes) did not affect facilitation.

Fiber volley (FV) and EPSP amplitudes were measured with a minimum-to-baseline (MTB) algorithm and compared with a measure of the rate of change for the initial waveform slope (*dV/dt*). Figure 1A shows an averaged EPSP with the two methods represented by dashed lines (MTB) and solid lines (*dV/dt*, slopes). The two measures were compared for variability by linear fits when plotted as functions of the stimulus voltage over a 3 V range (Fig. 1A, *right panels*). In both cases the data obtained by the MTB algorithm had a higher correlation coefficient than the slope data, especially at low voltages as in the case of FVs. This trend is also evident in Fig. 1A, (*lower left*) that shows a greater scatter of slope measurements at lower stimulus voltages at the start and end of the experiment. Facilitation was plotted in the same panel (solid line) and remained constant across large changes in EPSP amplitudes.

Isoflurane produced a reversible depression of EPSP amplitudes, accompanied by an increase in facilitation as previously reported [12,21,22]. Figure 1B shows the time course of a typical experiment in which 350 μM isoflurane (1.5 vol %; 1 MAC) depressed EPSP amplitudes. The conditioning (1st) EPSP was depressed by ~60%. The test EPSP (2nd) was depressed by a smaller amount, which increased facilitation by ~20% (n = 5). The actions of isoflurane on glutamatergic neurotransmission follow the same pattern as when extracellular Ca^2+ ^is reduced from 2 mM to 0.8 mM (Fig. 1C). Low external Ca^2+ ^depressed both conditioning and test EPSPs while facilitation increased. By contrast, TTX (60 nM; Fig. 1D) depressed EPSPs to about the same level as isoflurane or low Ca^2+^, however TTX did not change facilitation.

**Figure 2:**
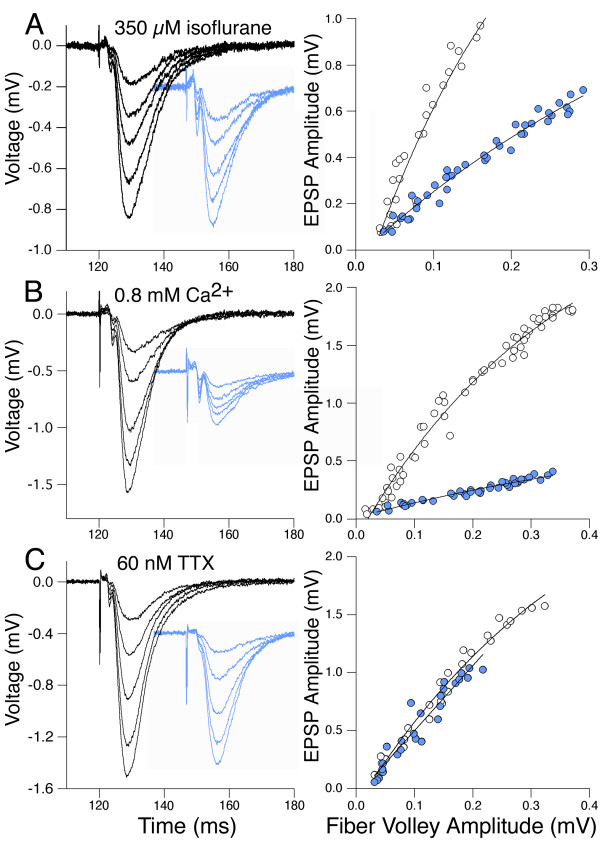
**Depressant actions on FV-EPSP relations**. *A*, (*Left*) Isoflurane (350 μM; 1.5 vol %) reduced EPSP amplitudes over a range of stimulus levels. Control responses are shown in black and isoflurane-depressed EPSPs are in blue. (*Right*) Isoflurane reduced the slope of the FV-EPSP relation (blue circles). *B*, (*Left*) EPSP amplitudes were strongly decreased when external Ca^2+ ^was reduced from 2 mM to 0.8 mM. (*Right*) slope of the FV-EPSP relation was reduced by 0.8 mM Ca^2+ ^(blue circles). *C*, (*Left*) reduction of EPSP and FV amplitudes by tetrodotoxin (TTX; 60 nM). (*Right*) no change in the FV-EPSP relation in the presence of TTX. All FV-EPSP relations were well fit by single exponential functions.

Isoflurane depressed glutamatergic EPSPs over a wide range of stimulus voltages (Fig. 2A). EPSPs uniformly decreased in amplitude after exposure of the slice to 350 μM isoflurane (inset). Figure 2A (right) shows the relation between FV and EPSP amplitudes. FV-EPSP relations were obtained over a 5 V stimulus range in 0.2 V increments. The results show isoflurane depressed EPSPs independently of the FV amplitudes (Fig. 2A, *right*). Isoflurane shifted the apparent slope of the FV-EPSP relation (Fig. 2A, blue circles), which would be expected for depression of EPSP amplitudes with no or little action on FV amplitudes. This trend was also observed for experiments with reduced external Ca^2+ ^(0.8 mM) in which EPSPs were reduced in amplitude and the FV-EPSP slope was shifted in a similar manner (Fig. 2B). However, the FV-EPSP relation obtained with TTX (60 nM) was entirely dependent on FV amplitudes (Fig. 2C). While EPSPs were depressed by TTX, the FV-EPSP slope relation in the presence of TTX was unchanged (Fig. 2C, *right*) since all of the EPSP depression produced by TTX was due to depression of FV inputs.

**Figure 3:**
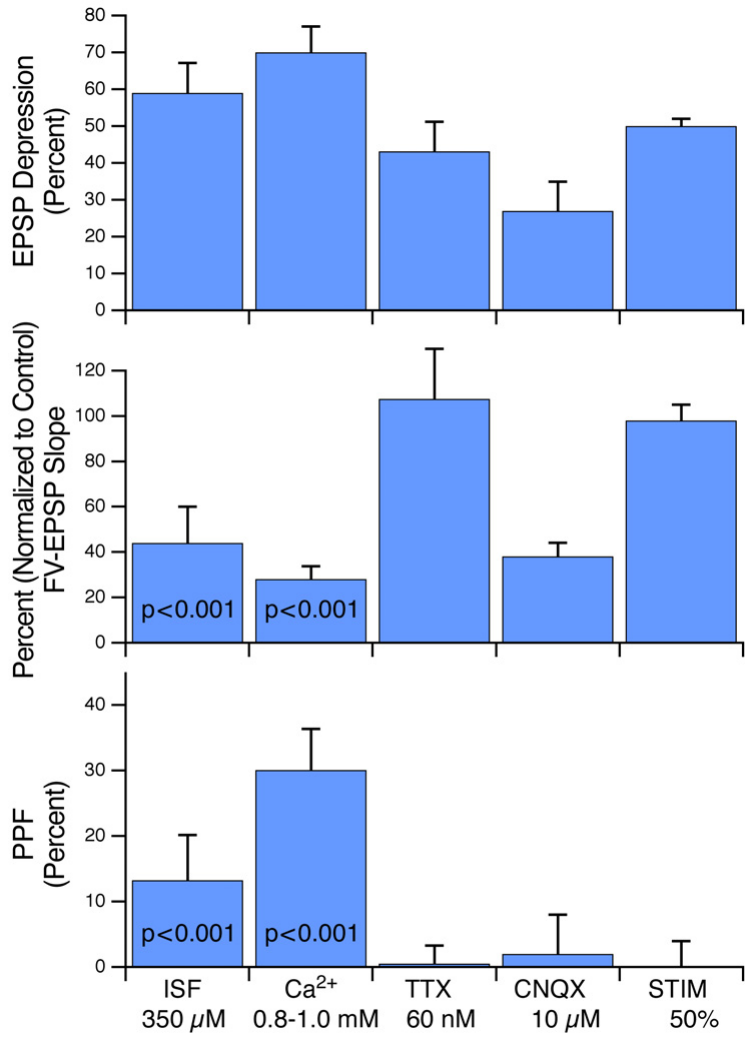
**Actions of isoflurane (ISO, 1 MAC), low extracellular Ca^2+ ^(0.8 – 1.0 mM), TTX (60 nM), CNQX (10 μM) and 50% stimulus (STIM) voltage**. Experiments with 0.8 mM and 1.0 mM external Ca^2+ ^were pooled, as the changes in facilitation and EPSP depression under each condition were almost identical. The actions of these treatments are shown for EPSP depression (*top panel*), FV-EPSP curve slopes (I/O slopes; *middle panel*) and paired-pulse facilitation (PPF; *lower panel*). Each bar represents the mean ± standard deviation for at least five experiments from different slice preparations. Note that each treatment substantially depressed EPSPs but only isoflurane and low Ca^2+ ^produced a significant change in the I/O slope compared to TTX (p < 0.001 in both cases, ANOVA). PPF with isoflurane and low Ca^2+ ^was also significantly greater than with TTX (p < 0.001 and p < 0.001 respectively, ANOVA).

Figure 3 shows the results of these experiments summarized in a bar graph. Also shown are experiments involving perfusion with 10 μM CNQX or reduced stimulus voltage. The FV-EPSP slope (middle panel) was reduced significantly by isoflurane and low external Ca^2+ ^compared with TTX (p < 0.001 in each case, ANOVA). Depression of FV-EPSP slopes suggests an effect on excitation-release coupling. On the other hand CNQX, an AMPA/kainate receptor antagonist, also reduced the FV-EPSP slope, presumably by a postsynaptic action. Reduction of the stimulus voltage by 50% left the FV-EPSP slope unchanged, which is consistent with an exclusively axonal action and comparable to TTX treatment (Fig. 3, middle panel).

Isoflurane significantly increased paired-pulse facilitation (p < 0.001) as did low Ca^2+ ^(p < 0.001) when compared with TTX (ANOVA, Fig. 3, *lower panel*). By comparison, facilitation was largely unaffected by 10 μM CNQX or reduced stimulus voltage. Isoflurane (350 μM) depressed the first EPSP by about the same amount as when external Ca^2+ ^was reduced to 0.8 mM (Fig. 3*, top panel*, no significant difference, ANOVA, n = 6). However, in the same experiments (Fig. 3, *lower panel*), isoflurane's action to enhance facilitation was significantly less than low Ca^2+ ^(p < 0.001, ANOVA, n = 6). Because facilitation is increased when synaptic release probability is reduced [23,24], the actions of isoflurane appear to involve additional sites besides those related to excitation-release coupling or Ca^2+ ^ion influx.

### Partial reversal of isoflurane depression of EPSPs by high external calcium

**Figure 4:**
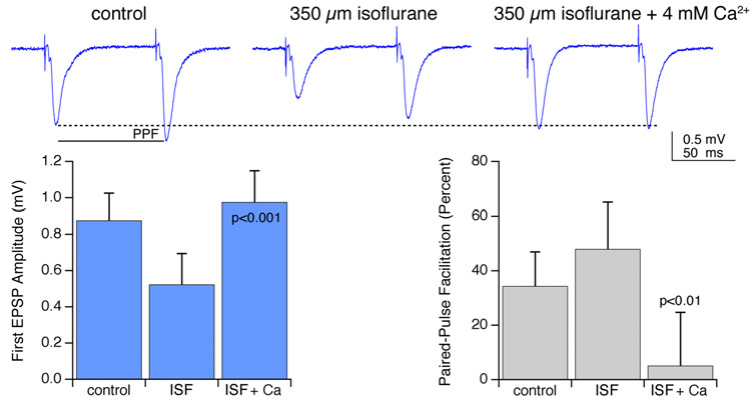
**High external calcium partly reverses isoflurane depression of EPSPs**. (*Top*) control EPSP pairs at left show the first EPSP amplitude (dashed line) and facilitation of the second EPSP (solid line). The center trace shows depression of both EPSPs in the presence of 350 μM isoflurane. The traces at right show reversal of isoflurane depression for the first EPSP and partly for the second in the presence of 350 μM isoflurane and ACSF with [Ca^2+^] doubled to 4 mM. (*Lower Left*) bar graph shows reversal of isoflurane (ISF) depression of the first EPSP. The first EPSP amplitude was depressed ~50% by isoflurane, but increased significantly when isoflurane was delivered in ACSF containing 4 mM Ca^2+ ^(ANOVA, p < 0.001, n = 6). (*Lower Right*) bar graph shows that facilitation tended to increase in the presence of isoflurane but was then significantly depressed when isoflurane was delivered in ACSF containing 4 mM Ca^2+ ^(ANOVA, p < 0.01, n = 6).

Since low external Ca^2+ ^produced a depressant effect like isoflurane on glutamatergic neurotransmission, we were interested in whether raising external Ca^2+ ^would reverse the actions of isoflurane. We recorded EPSPs during perfusion of isoflurane in ACSF with high Ca^2+ ^(4 mM). Figure 4 shows that isoflurane depression of the first EPSP was reversed in high Ca^2+ ^ACSF. The reversal was significant (Fig. 4, *lower left*). However, isoflurane delivered in high Ca^2+ ^ACSF still exerted a depressant action on the second EPSP and facilitation was significantly decreased from isoflurane in standard ACSF (Fig. 4, *lower right*).

### Minimal fiber volley and somatic Na spike depression by isoflurane

**Figure 5:**
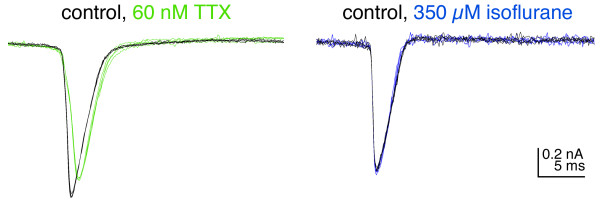
**Insensitivity of somatic spike currents to isoflurane**. Control traces at left are four overlaid leak-subtracted inward spikes. The records are from a representative whole-cell perforated patch in the pyramidal cell body layer of the hippocampal CA1 region. 100 ms voltage pulses to -30 mV were applied at 10 s intervals to elicit spikes (V_H _= -70 mV). Currents in green show four spikes recorded after a 15 min exposure to 60 nM TTX. At right are overlays of spike currents recorded before and after a 10 min exposure to 350 μM isoflurane (1 MAC). The blue traces were recorded after exposure to isoflurane.

Fiber volleys represent a summation of many action potentials that arrive at the CA1 region following Schaffer collateral stimulation. Due to the difficulty of patching nerve terminal fibers to study action potentials directly, we decided to study isoflurane actions on somatic Na currents recorded from whole cell patches on cells in the CA1 pyramidal cell layer. The perforated patch method allowed us to reliably obtain access to the cell interior while patching blind [25]. Currents were activated by a voltage step to -30 mV from a holding potential of -70 mV. A 100 ms depolarizing pulse was delivered every 10 s. Representative currents in Fig. 5, *left *show a decrease in amplitude and a slight delay after a 15 minute exposure of the slice to 60 nM TTX. In a separate experiment, currents recorded 10 minutes after exposure to 350 μM isoflurane were indistinguishable from controls (Fig. 5, *right*). These results are in general agreement with our field recording experiments, although somatic Na channels may have different properties than those in terminal fibers.

**Figure 6:**
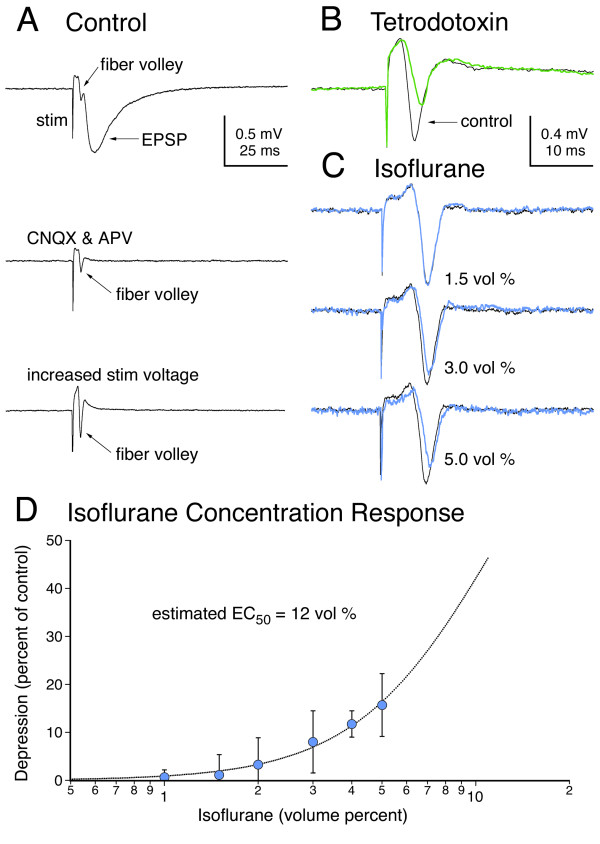
**Depression of FVs by isoflurane**. *A*, (*top*) An extracellular recording under control conditions showing the FV is partially occluded by the larger EPSP. *A*, (*lower*) The FV was isolated with CNQX (8.6 μM) and APV (125 μM) to block AMPA/kainate and NMDA receptors, respectively. *B*, Partial block of a FV by 60 nM TTX (green trace). *C*, Isoflurane produced small reductions of FV amplitudes (blue traces). *D*, Concentration dependence of the depression of FV amplitudes by isoflurane. Data were normalized to control conditions in the absence of drug. Each point represents the mean of multiple experiments carried out in different slice preparations (n = 6 each data point), with standard deviations represented by the error bars. The dashed line is a least-squares fit to a Boltzmann relation with an EC_50 _= ~12 vol % and a Hill coefficient of 2 (χ^2 ^= 2.5). All measurements were made following at least a 20 minute period for the perfusion system to equilibrate.

Because fiber volleys are partially occluded by EPSPs (Fig. 6A), the dose-dependent action of isoflurane on fiber volleys was investigated following block of glutamate receptors with 2-amino-5-phosphonovaleric acid (APV; NMDA receptor blocker, 125 μM) and 6-cyano-7-nitroquinoxaline-2,3-dione (CNQX; AMPA/kainate receptor blocker, 8.6 μM). This treatment eliminated EPSPs leaving only FVs (Fig. 6A, *lower panel*). Observable block of FVs only became apparent with high (3 vol %) or supraclinical (5 vol %) isoflurane (Fig. 6C). At these concentrations isoflurane produced a reduction of FV amplitudes in a manner comparable to, but not as great as 60 nM TTX (Fig 6B). FV amplitudes were depressed in a dose-dependent manner (Fig. 6D) with an EC_50 _of ~2.8 mM (12 vol. %; 8 MAC) estimated from a least-squares fit extrapolation. But at clinical levels, 350 μM (1 MAC) isoflurane only depressed FV amplitudes by 1.2 ± 4.3% (n = 8). This is in contrast to the volatile anesthetic halothane, which depressed FV amplitudes by ~18% at 1 MAC [9], using identical recording conditions.

## Discussion

Volatile anesthetics are known to depress glutamatergic synaptic transmission, although the molecular mechanisms are unclear. Wu et al. [15] reported that isoflurane inhibited presynaptic action potentials in the calyx of Held to reduce glutamatergic neurotransmission. We were interested in further study of the presynaptic action of isoflurane, as Wu et al. [15] did not compare isoflurane and TTX effects, nor did they look at effects on facilitation. To test the generality of isoflurane depression of action potentials, we studied the effects of isoflurane in comparison with TTX on EPSPs in the CA1 region of the rat hippocampus and on FVs recorded from presynaptic Schaffer-collateral fibers. TTX blocks all brain Na channels, including those in the calyx of Held [26,27]. We found that clinical concentrations of isoflurane had little to no inhibitory action on FV amplitudes, although EPSPs were strongly depressed and facilitation was increased. Somatic Na currents were also unaffected by 350 μM isoflurane. On the other hand, TTX reduced FV amplitudes and EPSP amplitudes but had no effect on facilitation. TTX also had no effect on the slope of the FV-EPSP relation. The actions of TTX likely resulted in part from nerve fiber conduction block. Fewer conducting fibers would reduce responsiveness to the stimulating electrode and account for the similar actions of TTX and reduced stimulus voltage on paired-pulse facilitation and the FV-EPSP relation (Fig. 3). TTX could also reduce Na spike amplitudes and decrease fiber conduction velocity [28] (Fig. 6B).

Unlike TTX, isoflurane at clinical concentrations doesn't appear to inhibit Na channel activity in hippocampal Schaffer-collateral fibers. Depression of EPSPs and enhancement of facilitation by isoflurane likely results from actions downstream from Na channels at sites that contribute more directly to excitation-release coupling, as supported by the insensitivity of the FV-EPSP slope to TTX (Fig. 2).

Fiber volleys in Schaffer collateral fibers represent a summation of closely-timed multiple action potentials. Their close temporal coordination is reflected by the short duration of the fiber volley, which is typically about 5–8 ms. FV-EPSP relations typically show an asymptotic decrease at higher FV amplitudes (Fig. 2, *right panels*), which contrasts to the accelerating relation of the action potential – EPSC relation in the calyx of Held [15]. However, Wu et al [15] obtained their EPSC data by use of a scaled action potential as a voltage command while we obtained EPSP data by changing the stimulus voltage. Our methodology reflects an experimental focus on the properties of a population of synapses, with results that show a broad insensitivity of terminal fiber Na channels to isoflurane. Further study is needed at individual CA1 synapses to determine whether a subpopulation may be more sensitive to conduction block by isoflurane.

Elevated external Ca^2+ ^reversed isoflurane depression of the first EPSP. This is likely related to the enhanced release of transmitter by increased Ca^2+ ^influx during nerve terminal depolarization. Elevated external Ca^2+ ^may also enhance NMDA receptor currents. However, this contribution to the EPSP is likely to be small, as the proportion of the NMDA receptor current carried by Ca^2+ ^is fractional, e.g. 17% of the total NR1/NR2A current [29].

Voltage-gated P/Q type Ca channels are localized at synaptic terminals in many central neurons [30], including the mature calyx of Held where they closely associate with synaptic vesicles [31] and appear to have an important role in paired-pulse facilitation [32]. High external [Ca^2+^] will increase Ca^2+ ^influx when Ca channels open in response to depolarization. The result would likely increase the synaptic release probability [33] and tend to reduce facilitation, as we observed (Fig. 4). While P/Q Ca channels are not strongly inhibited by volatile anesthetics [19] there remains the possibility that volatile anesthetics may reduce calcium-induced calcium release from internal stores, as described in dendrites of hippocampal pyramidal cells [34] and suggested as an anesthetic mechanism for pentobarbitone [35].

Another possible site includes the *N*-ethylmaleimide-sensitive factor attachment protein receptor (SNARE) complex. The SNARE complex is involved in vesicle docking and fusion at most synapses. Disruption of SNARE proteins with Botulinum toxins A and F can each reduce the initial release probability. However, Botox F doesn't produce facilitation unless external [Ca^2+^] is lowered [36]. Our results showing partial reversal of isoflurane effects on neurotransmission with high external [Ca^2+^] suggest the SNARE complex may warrant further study as targets of volatile anesthetics. In fact, isoflurane and halothane have recently been reported to bind to syntaxin and the SNARE complex at clinical concentrations [37], although the binding sites on the SNARE proteins have not yet been characterized.

It is important to note that not all volatile anesthetics may target the same molecular sites. Our results with isoflurane differ from the reported actions of halothane on synaptic transmission. Like isoflurane, halothane depresses EPSP amplitudes in rat hippocampal slices at clinical concentrations [13]. However, halothane also depresses Schaffer-collateral axon fiber volley amplitudes [9]. Unlike TTX, halothane has no effect on conduction velocity but does increase facilitation of EPSPs [9]. While the dissimilar pharmacological profiles for halothane and isoflurane are inconsistent with a unitary model of anesthesia, both can be understood within a multi-site model of volatile anesthetic action [38-40].

The weak depressant action of isoflurane on Schaffer-collateral FVs indicates that presynaptic actions of isoflurane are not homogeneous in the brain – especially when contrasted to the results reported by Ouyang et al. [16] and Wu et al. [15]. Furthermore, Baudoux et al [35], who studied Ca^2+ ^transients in CA1 pyramidal cell axons, concluded that general anesthetics are unlikely to interfere with action potential propagation even in fine axon collaterals. Factors that could affect regional variability in action potential depression include the degree of terminal fiber myelination [cf. [8]; although see [5]] as well as the diversity of voltage-gated Na channel expression and subcellular distribution [41]. Five TTX-sensitive Na channel α-subunits have been reported to be expressed in hippocampal neurons: Na_V_1.1, Na_V_1.2, Na_V_1.3, Na_V_1.6 and Na_V_1.7 [42], with complex patterns of expression even between different neurons. On the other hand, Leão et al. [27] reported that Na channels are absent from the calyx of Held. Their immunocytochemical data show Na_V_1.6 but not Na_V_1.2 was expressed at the axonal heminodes from which action potentials passively invade the calyx [27,43]. These data taken together suggest that the expression pattern of Na channel α-subunits in neurons can influence anesthetic sensitivity. In addition, α-subunits are frequently associated with a β1 subunit that can modulate Na channel gating [44]. So it appears that there are numerous possible isoforms of Na channels that are expressed heterogeneously in the CNS. The degree to which these isoforms differ with respect to volatile anesthetic sensitivity is presently unknown.

## Conclusion

We conclude that for glutamatergic hippocampal neurons, isoflurane depresses transmission downstream from Na channels. Unlike TTX, isoflurane simultaneously increased facilitation and depressed EPSPs with only a minimal reduction of fiber volley amplitudes. Consistent with previous studies, a likely mechanism of isoflurane action is to reduce presynaptic Ca^2+ ^levels following nerve terminal depolarization and/or to disrupt the vesicle release process.

## Methods

The experimental protocols were approved by the Institutional Animal Care Committee at Stanford University and adhered to the published guidelines of the NIH, the Society for Neuroscience and The American Physiological Society. Male Sprague-Dawley rats were anesthetized with diethyl ether (22 vol. % in air). The animals were killed by decapitation. Brains were rapidly removed and placed in ice-cold (5°C) artificial cerebrospinal fluid (ACSF) that was pre-gassed with carbogen (95%-O_2_/5%-CO_2_). The ACSF had the following composition (in mM): 124 NaCl, 3.5 KCl, 1.25 NaH_2_PO_4_, 26 NaHCO_3_, 2 CaCl_2_, 2 MgSO_4_, and 10 D-glucose. Whole brain coronal slices (400 μm) were cut with a Vibratome Model 1500 (Technical Products International, Inc., O'Fallon, MO). The brain slices were equilibrated for at least one hour at room temperature (22°C) in a humidified chamber partially filled with ACSF and continually bubbled with carbogen. Individual slices were transferred to a recording chamber, submerged in ACSF and equilibrated for an additional 10 minutes prior to electrophysiological recording. All experiments were conducted at room temperature. Brain slices were continuously perfused with oxygenated ACSF at a flow rate of ~1.5 ml/min.

The inhalation anesthetic isoflurane was mixed with carrier gas (95%-O_2_/5%-CO_2_) in a calibrated commercial vaporizer (Fluotec 3, Fraser Harlake, Orchard Park, NY) and bubbled into the ACSF reservoir that perfused the brain slices. This system has been previously calibrated for delivery of volatile anesthetics [45]. In some experiments the perfusion solution included 2-amino-5-phosphonovaleric acid (APV; NMDA receptor blocker, 125 μM) and 6-cyano-7-nitroquinoxaline-2,3-dione (CNQX; AMPA/Kainate receptor blocker, 8.6 μM) to block EPSPs.

Bipolar tungsten microelectrodes were placed on Schaffer-collateral fibers to electrically stimulate inputs to hippocampal CA1 pyramidal neurons. Glass micropipettes (Garner Glass, Claremont, CA) were pulled with a resistance of 2–5 MΩ, filled with ACSF and placed in stratum radiatum to record stimulus-evoked fiber volleys and excitatory postsynaptic potentials. Paired stimulus pulses (3–6 V, 300 μs duration; 80 ms interpulse interval) were delivered by a Grass stimulation isolation unit from a two-channel stimulator at a stimulus rate of 0.05 Hz. The field potentials were amplified, low-pass filtered at 20 kHz and sampled at 25 μs.

All responses were measured with DataWave or PClamp software and analyzed with IGOR Pro (WaveMetrics, Lake Oswego, OR). Fiber volley and EPSP amplitudes were measured from peak negative voltage to baseline (minimum-to-baseline) or alternatively, their initial slopes were measured between 20% and 80% of minimum. All responses were normalized as a percent of control, based on average amplitudes from a 20 min recording immediately before drug perfusion. We used InStat 3 (GraphPad Software, Inc.) to compare anesthetic effects between groups of time-matched experiments (two-way analysis of variance; ANOVA).

### Perforated patch recordings

Whole cell currents were recorded from patches in the pyramidal cell body layer of the CA1 region of hippocampal slices. Slices were perfused with ACSF as described above. Patch electrodes were pulled from Garner KG-33 glass. The recording micropipette resistances ranged from 4–6 MΩ and seal resistances ranged from 1–5 GΩ. The patch electrode-filling solution contained (in mM) 140 potassium gluconate, 10 HEPES, 0.2 EGTA, 2 MgCl_2_, 0.10 CaCl_2_, 2 Na_2_-ATP, 0.3 Na-GTP (pH 7.4; free Ca^2+ ^~100 nM). To obtain perforated patches, a stock solution of 100 mM amphotericin B (Sigma-Aldrich, St. Louis, MO) was prepared in DMSO and stored at -20°C. The stock solution was diluted (1/1000) into the pipette solution and sonicated for ~30 s to obtain a final amphotericin B concentration of 100 μM. Before seal formation, the voltage offset between the patch electrode and the bath solution was adjusted to produce zero current. Whole cell currents were recorded with an Axon Instruments MultiClamp 700A amplifier and PClamp software (Molecular Devices, Union City, CA). All experiments were performed at room temperature (~21–23°C).

## Authors' contributions

BDW carried out the electrophysiological studies, statistical analysis and drafted the manuscript. MBM participated in the electrophysiological studies, study design and helped to draft the manuscript. All authors read and approved the final manuscript.
